# Supplementary material for: Cytotoxicity Effect of Constituents of Pinus taiwanensis Hayata Twigs on B16-F10 Melanoma Cells
Source: Molecules. 2022 Apr 23;27(9):2731. doi: 10.3390/molecules27092731 (PMC9103300; doi:10.3390/molecules27092731)
Supplement: Supplementary file 1 [file molecules-27-02731-s001.zip › molecules-1661382-supplementary.pdf]

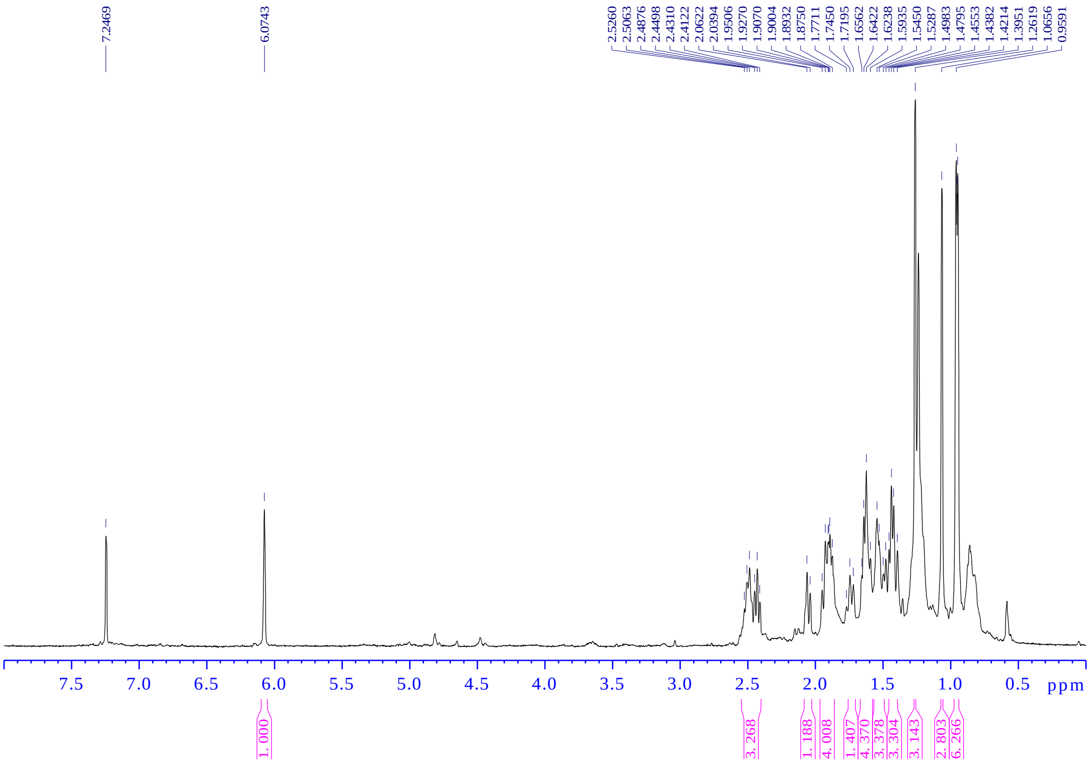

Figure S1： ^{1}H NMR spectrum of compound **3** (500 MHz in Chloroform-*d*)

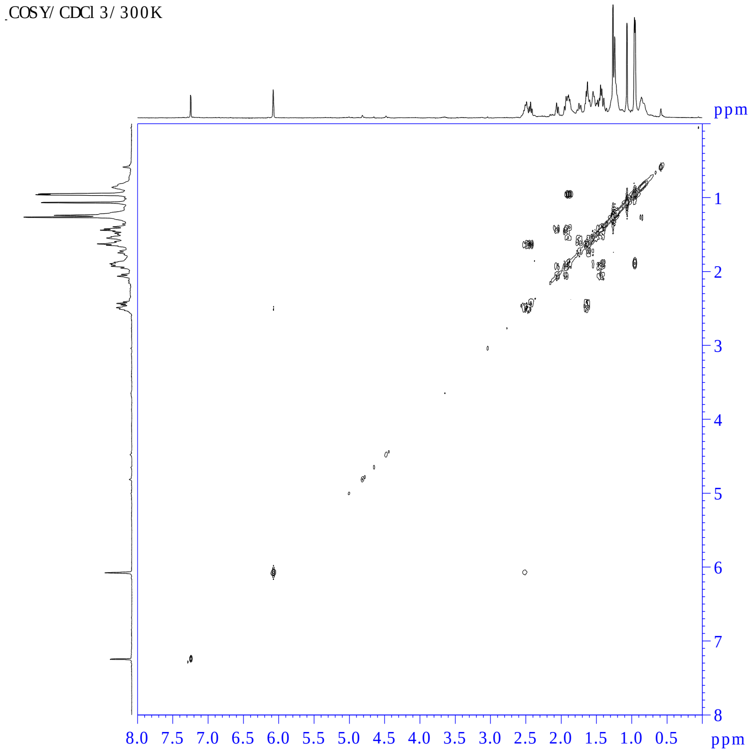

Figure S2: 2D COSY spectrum of compound **3** (Chloroform-*d*)

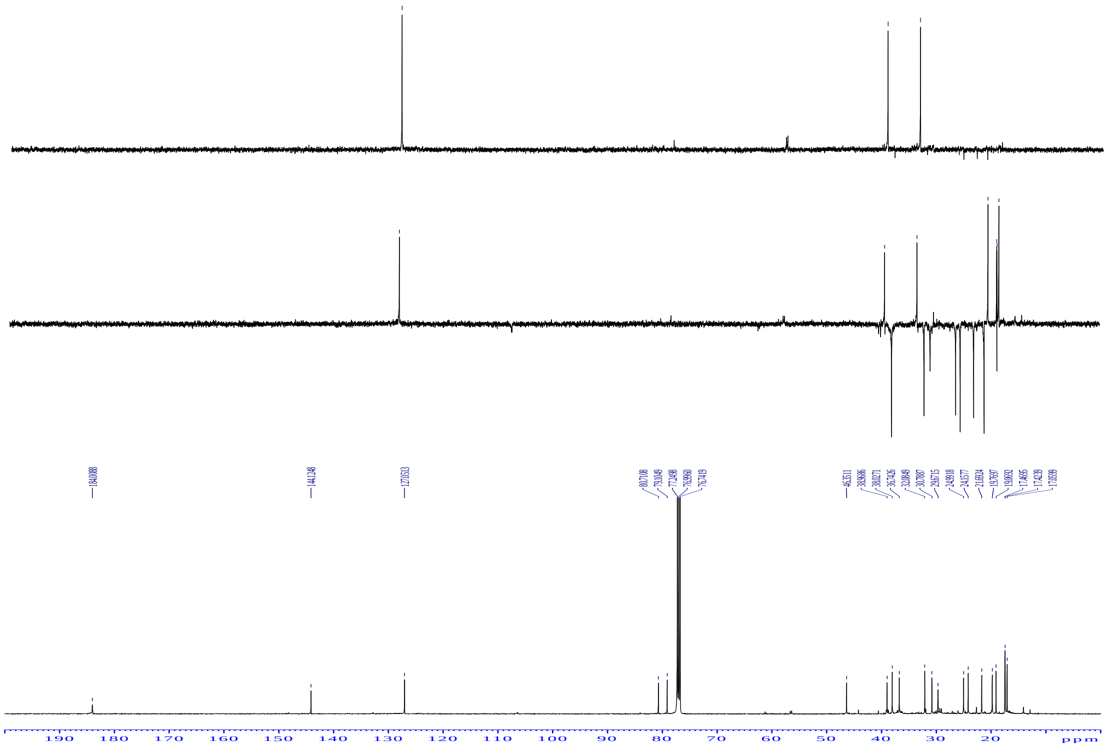

Figure S3: ^{13}C NMR spectrum of compound **3** (125 MHz in Chloroform-*d*)

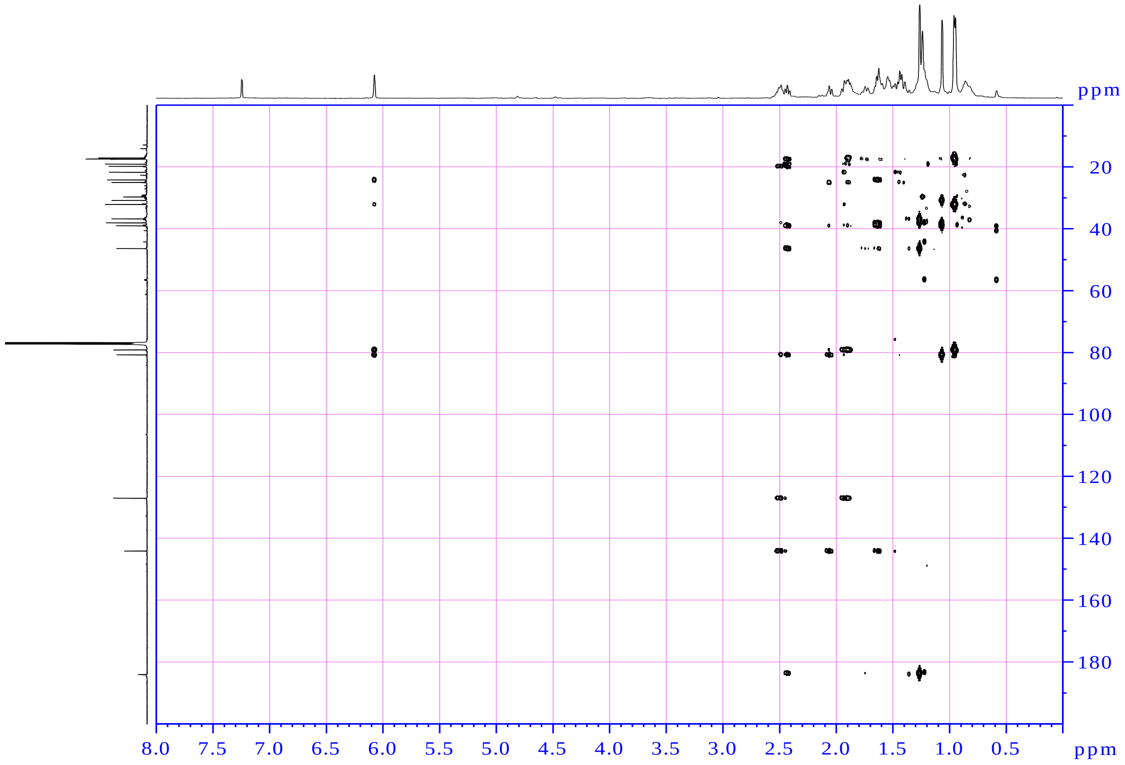

Figure S4: 2D HMBC spectrum of compound **3** (Chloroform-*d*)

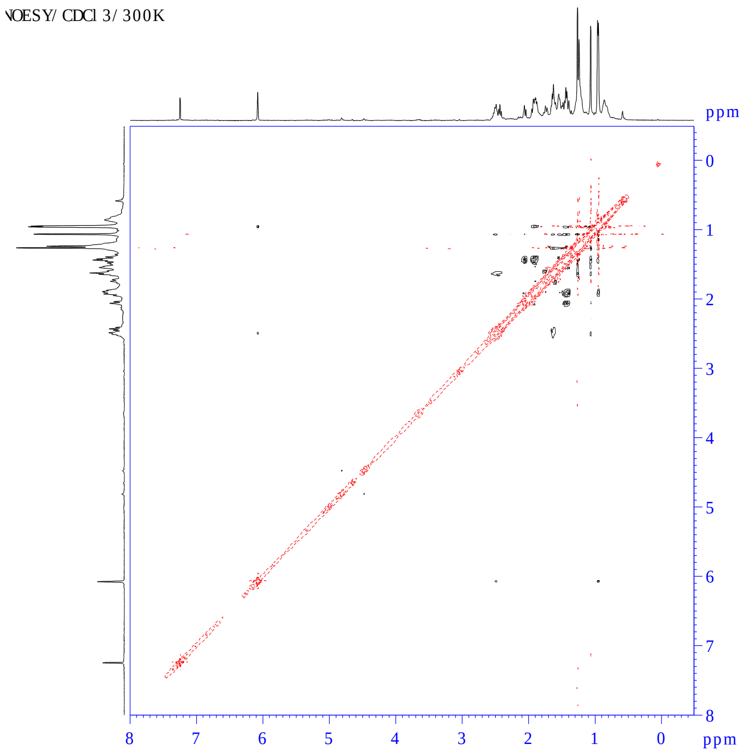

Figure S5: 2D NOSEY of compound **3** (Chloroform-*d*)
